# A Large Dussand Microphonograph

**Published:** 1898-04

**Authors:** 


"A LARGE Dussand microphonograph, now being constructed for the Paris exhibition of 1900, is expected to make the voice heard by 10,000 people," says *The Electrical Review*. "This form of apparatus is especially designed for the deaf, and for the study of the feeble sounds given out by the organs of the body in health and disease. It magnifies the voice much as a lens magnifies objects to the eye. The register is a modified phonograph, with a diaphragm vibrated by small electromagnets, receiving currents through a microphone; the repeater is some what similar, with a microphone attached to the membrane, the current for this being obtained from one to sixty battery cells and thence passing to a telephone. The intensity depends upon the amount of current passing. The instrument is being used in the education of deaf-mutes, and has had a marked effect in stimulating the nerves and apparatus of hearing."

